# Supplementary figures and images for: Fascin actin-bundling protein 1 regulates non-small cell lung cancer progression by influencing the transcription and splicing of tumorigenesis-related genes
Source: PeerJ. 2023 Dec 5;11:e16526. doi: 10.7717/peerj.16526 (PMC10704988; doi:10.7717/peerj.16526)

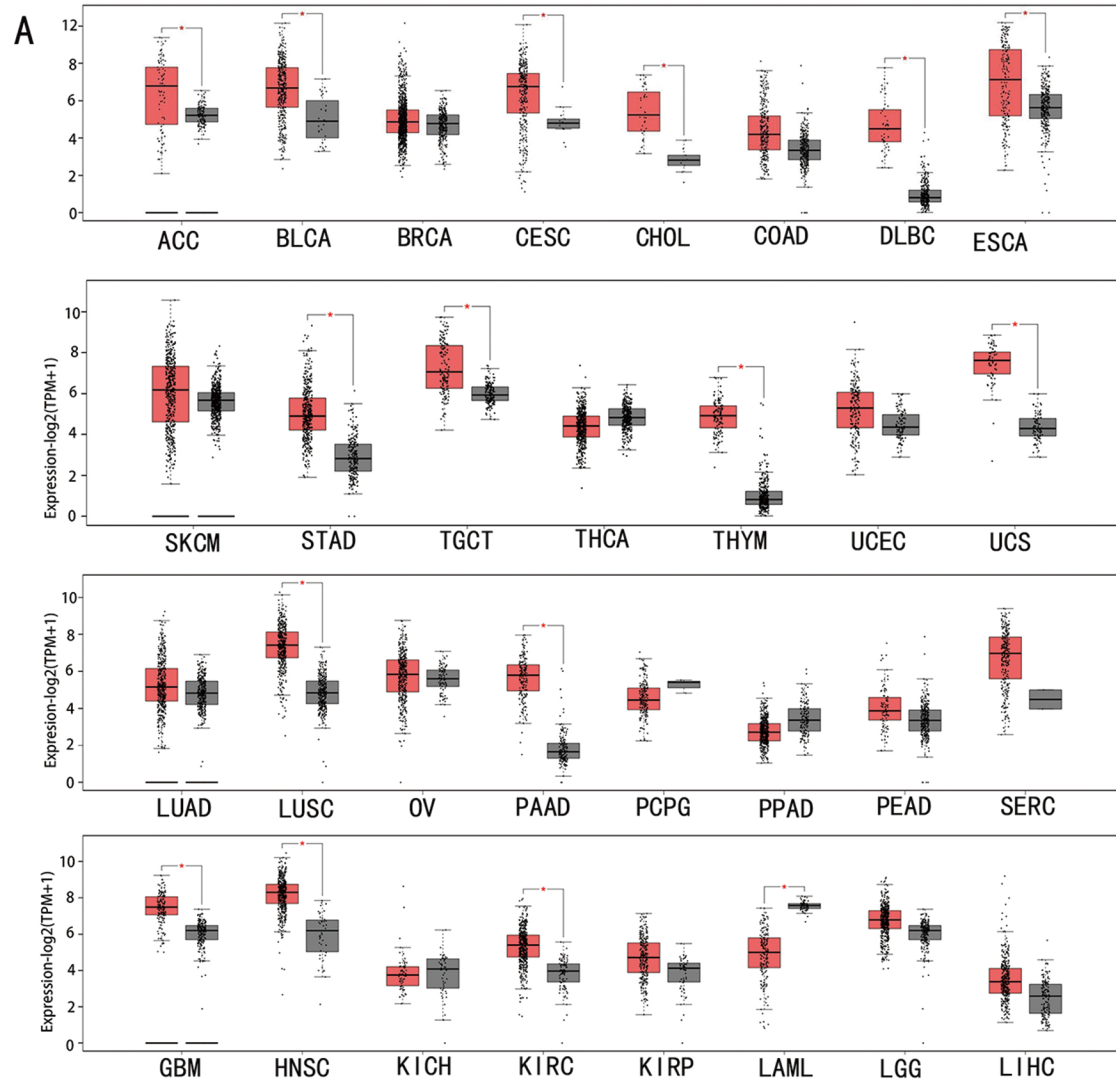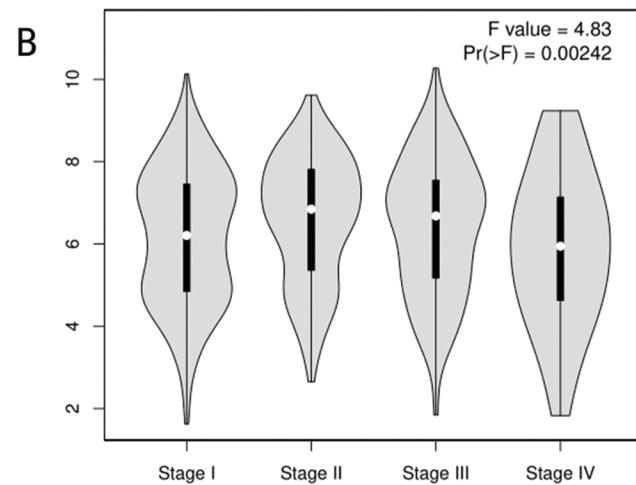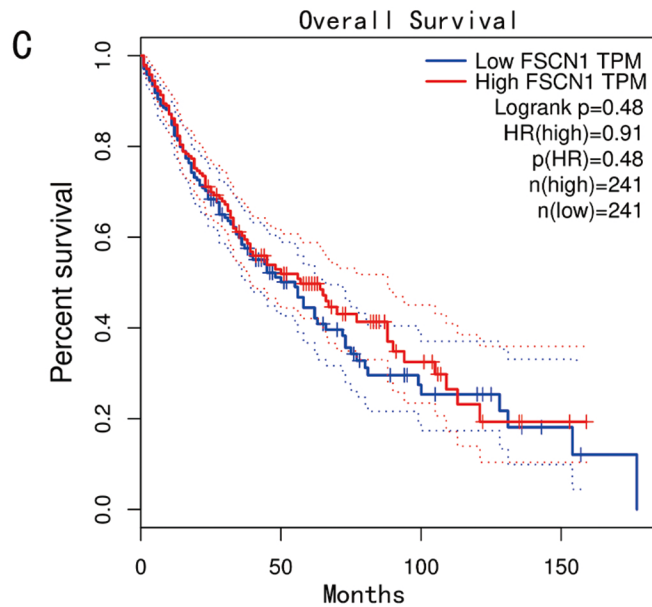

Supplement: Figure S1 — (A) Box plot of FSCN1 expression levels (TPM) in 36 tumor types from TCGA database *p-value < 0.05, unpaired Student’s t-test. The abbreviations of these tumor types are presented in TCGA database (https://www.cancer.gov). (B) Violin plot showing the change in expression levels of FSCN1 across the four disease stages in samples from patients with LUAD. Statistical analyses were performed using the one-way ANOVA method. (C) The Kaplan –Meier plot showing the difference in prognosis between LUSC patients with high and low FSCN1 expression levels. [file peerj-11-16526-s001.pdf]

A

## Disease-free survival

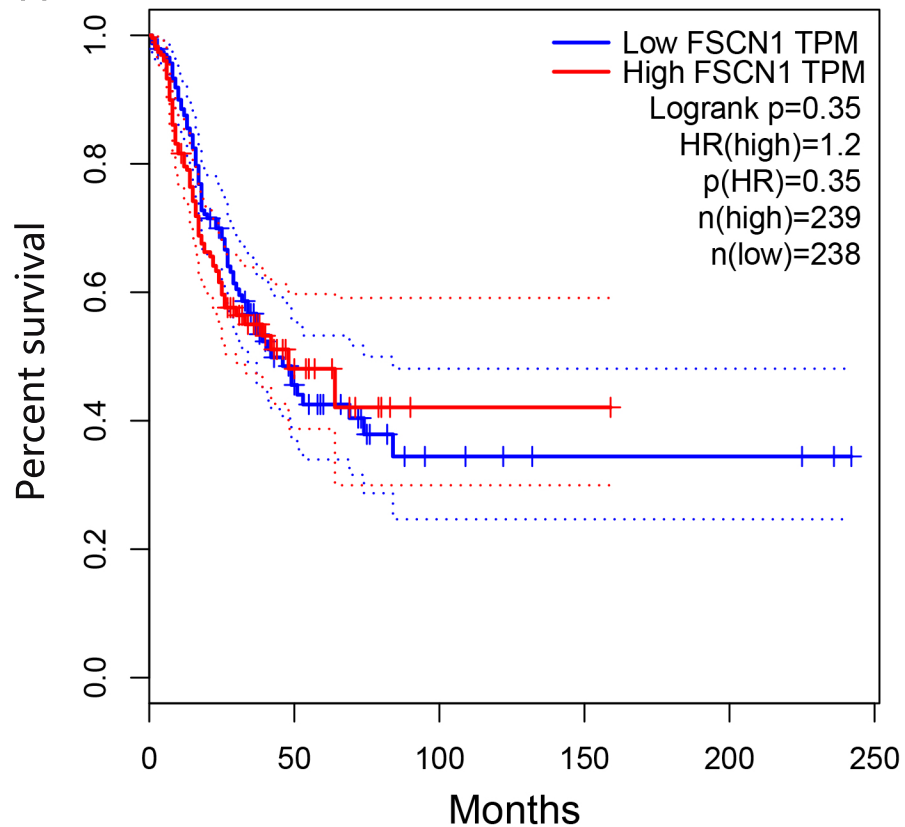

B

## Disease-free survival

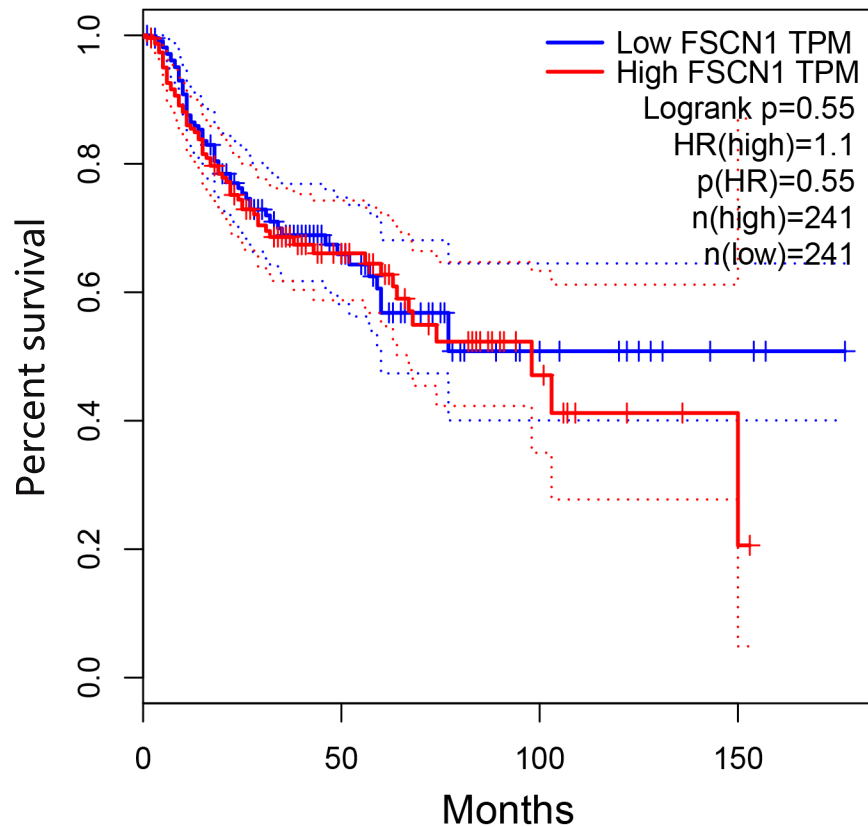

Supplement: Figure S2 — (P > 0.05). (A) Kaplan –Meier plot showing the disease-free progression difference between LUSC patients with high and low FSCN1 expression levels. (B) Kaplan–Meier plot showing the disease-free progression difference between LUAD patients with high and low FSCN1 expression levels. [file peerj-11-16526-s002.pdf]

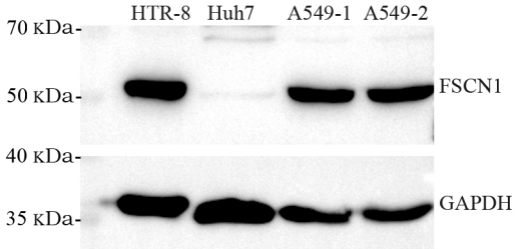

Supplement: Figure S3 [file peerj-11-16526-s003.pdf]

A

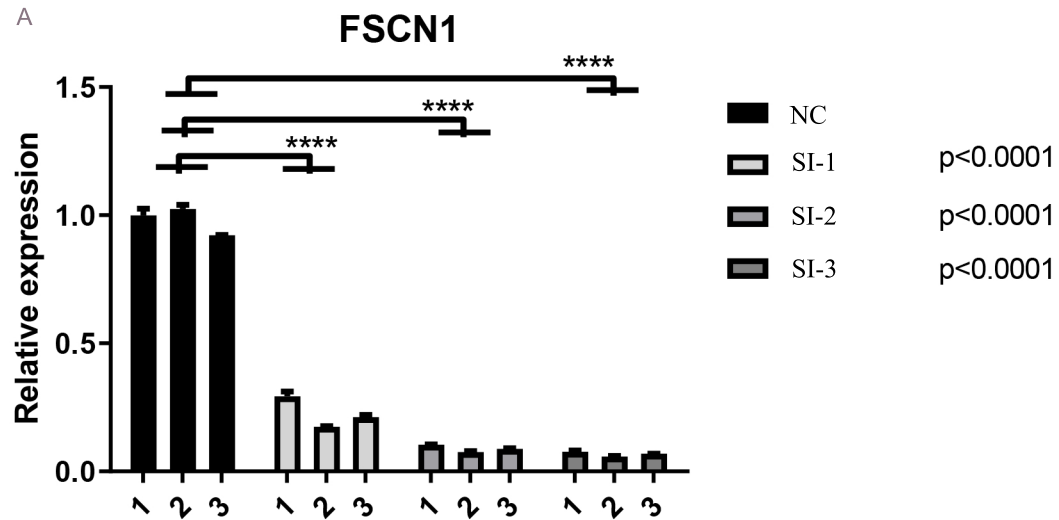

B

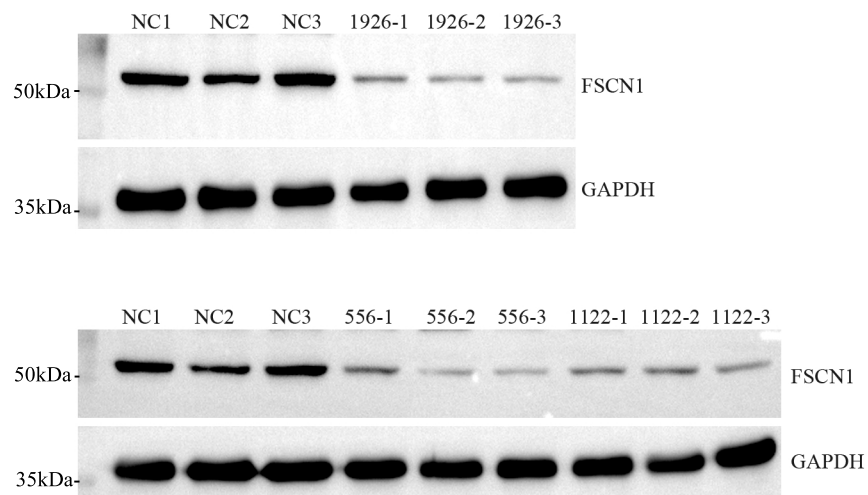

C

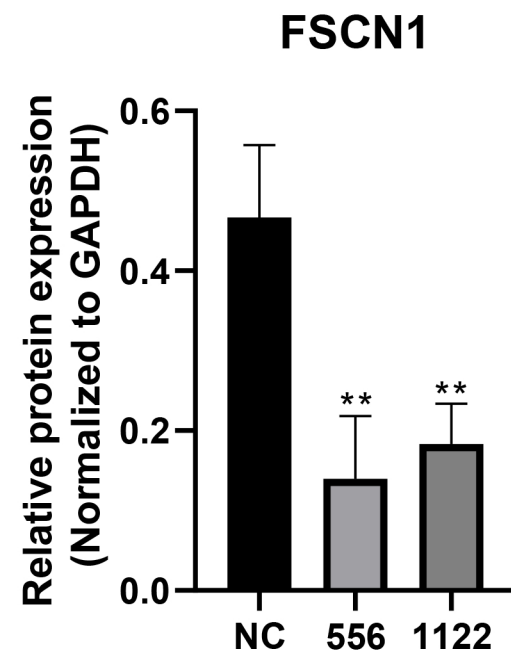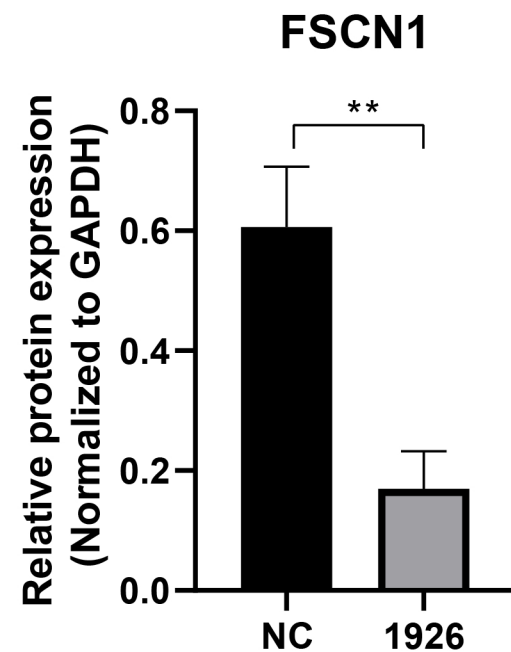

Supplement: Figure S4 — (A) The histogram shows the RT-qPCR results of NC and SI samples. Error bars represent mean ± SEM. (B–C) Western blotting results showed that the FSCN1 knockdown was successful. **P-value < 0.01 and ****P-value < 0.0001. NC, scramble siRNA (siNegative). SI-1, Si FSCN1-556.SI-2, Si FSCN1-1122.SI-3, Si FSCN1-1926. [file peerj-11-16526-s004.pdf]

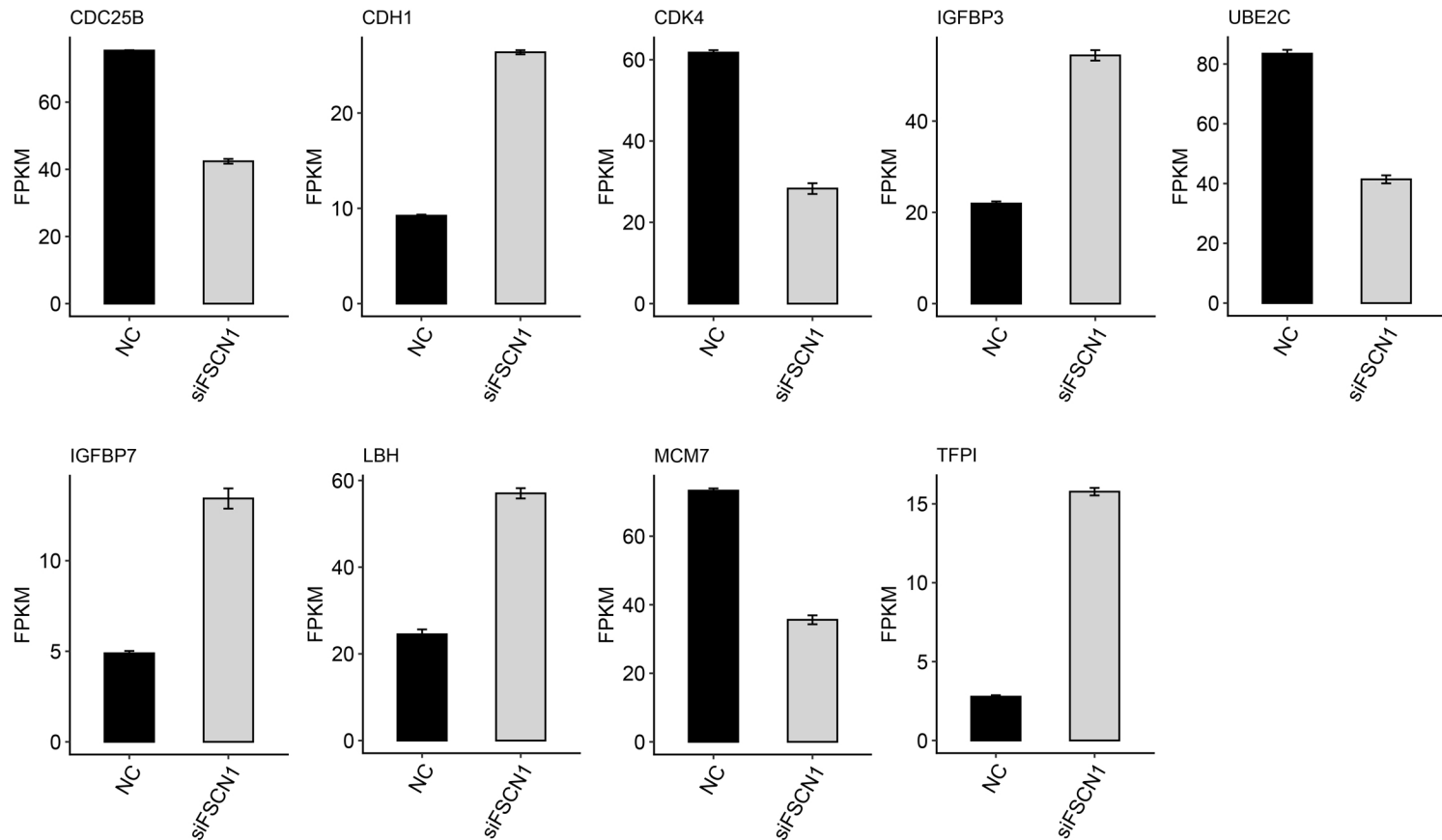

Supplement: Figure S5 — Bar plot showing the expression pattern and statistical difference of DEGs for specific key genes. Error bars represent mean ± SEM. ***P-value < 0.001. [file peerj-11-16526-s005.pdf]

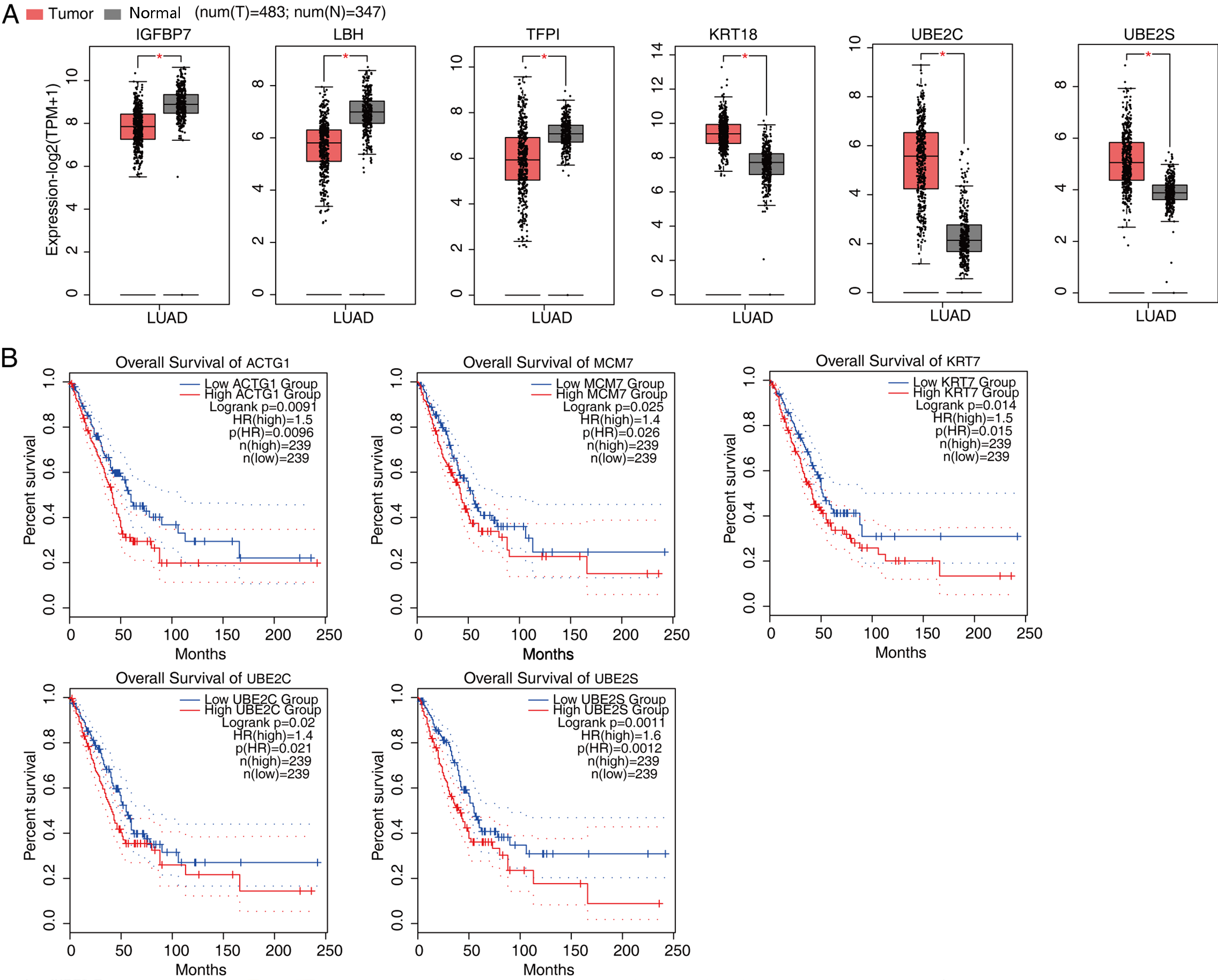

Supplement: Figure S6 — (A) Box plot showing the expression level of IGFBP7, LBH, TFPI, KRT18, UBE2C, and UBE2S in tumor and adjacent normal tissues of LUAD patients from TCGA based on the GEPIA2 database. (B) The survival curve representing outcomes associated with ACTG1, MCM7, KRT7, UBE2C and UBE2S expression in lung cancer based on data from TCGA database. [file peerj-11-16526-s006.png]

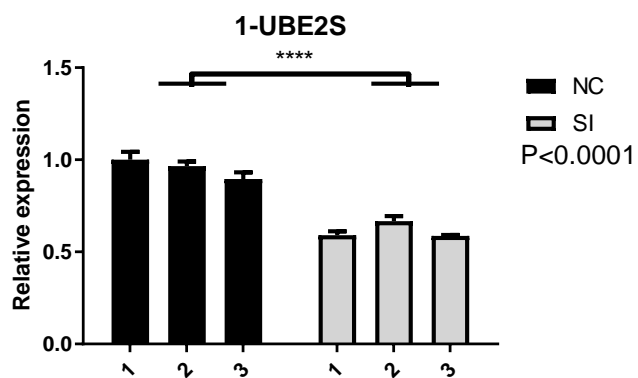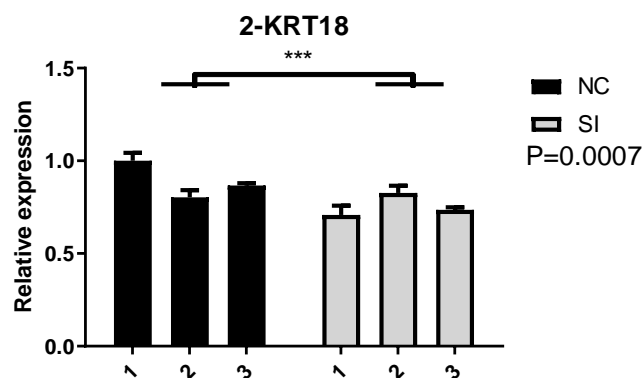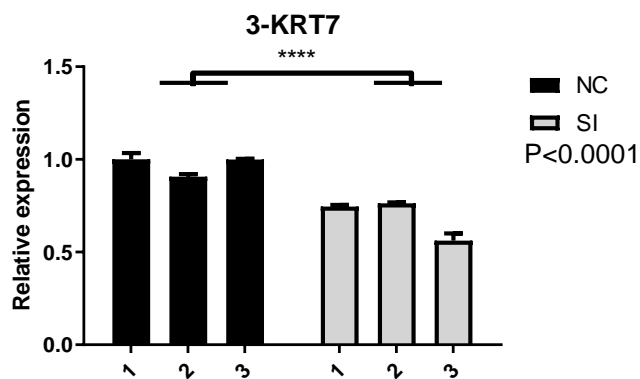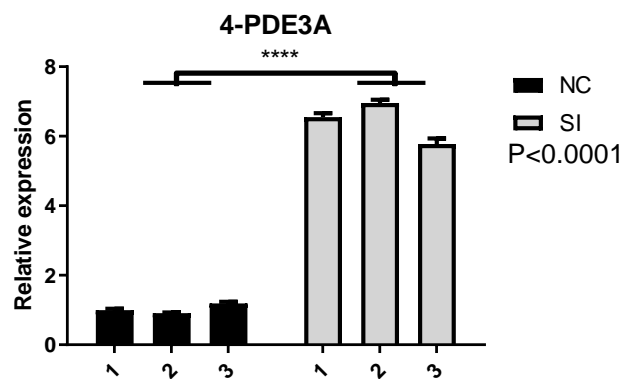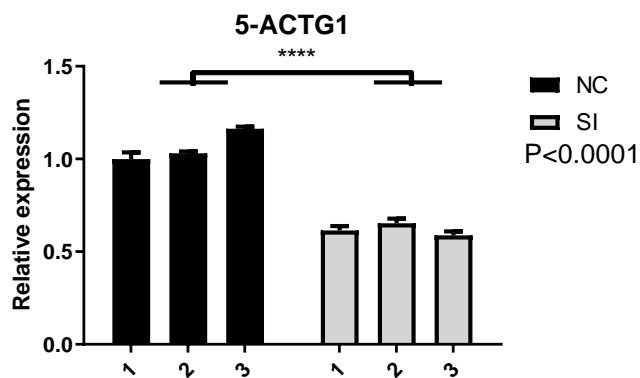

Supplement: Figure S7 — Black bars represent the control group, and grey bars represent FSCN1-silenced group. Error bars represent mean ± SEM. ***P-value < 0.001. ****P-value < 0.0001. [file peerj-11-16526-s007.pdf]

A

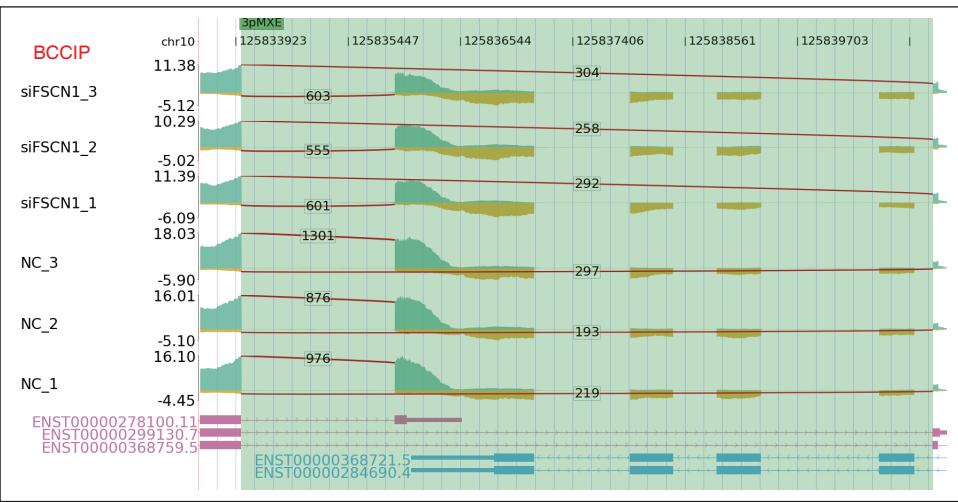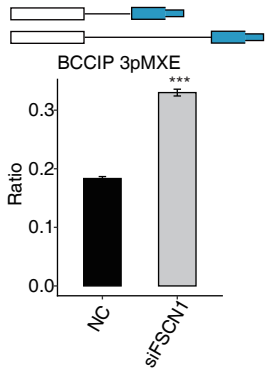

B

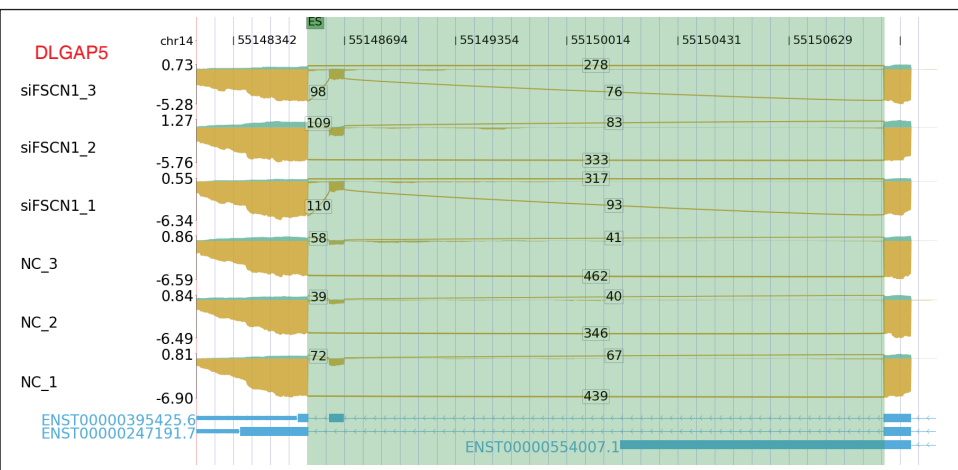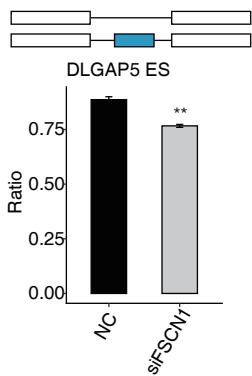

C

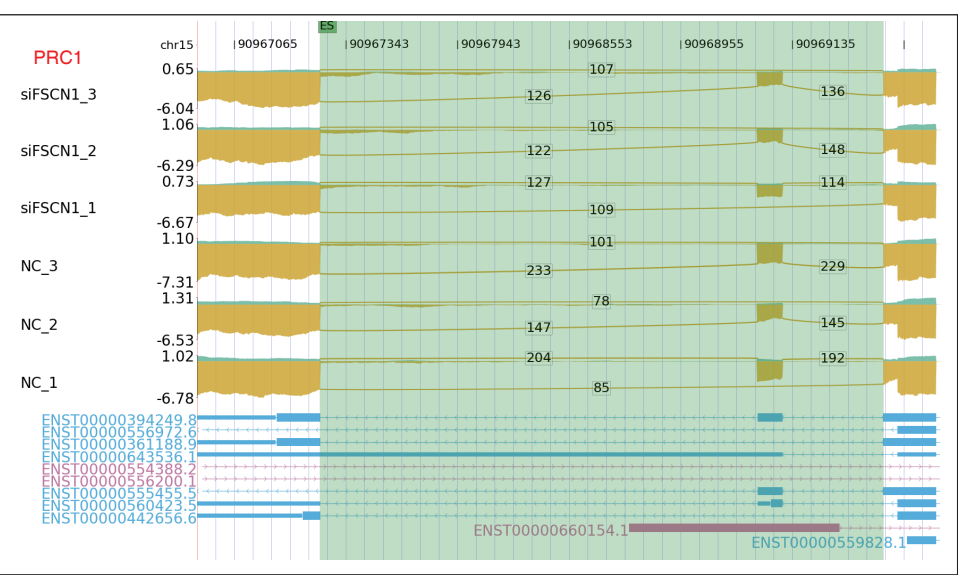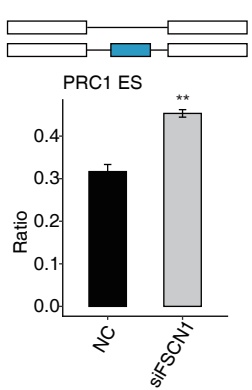

D

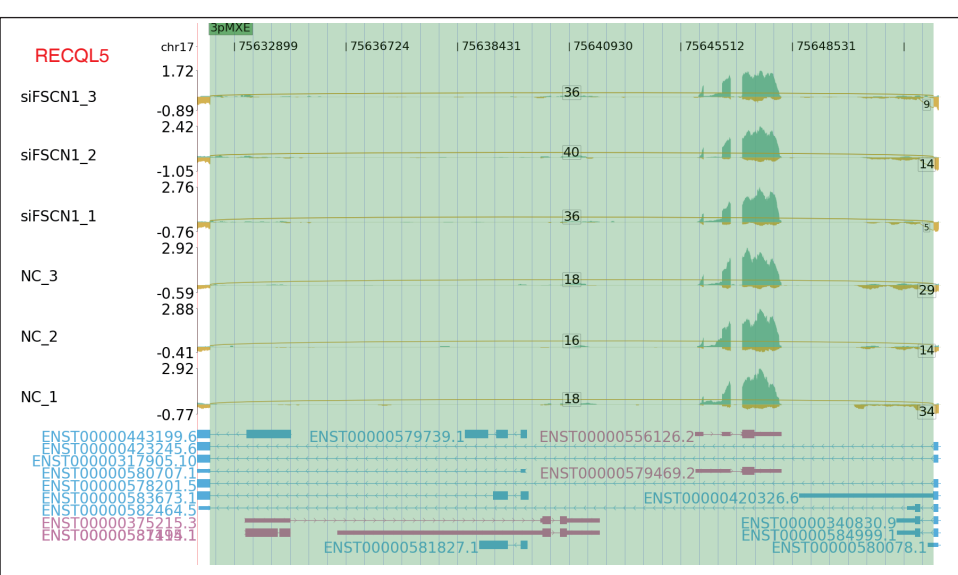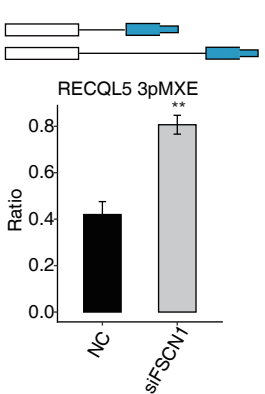

Supplement: Figure S8 — (A) FSCN1 regulates alternative splicing of BCCIP. Left panel: IGV-sashimi plot showing the regulated alternative splicing events (RASEs) and binding sites across mRNA. The read distribution of RASE is plotted in the top panel, and the transcripts of each gene are presented in the bottom panel. Right panel: The schematics depicting the structures of ASEs. RNA-seq validation of ASEs is shown at the bottom of the right panel. Error bars represent mean ± SEM. ***P-value < 0.001. (B) FSCN1 regulates alternative splicing of DLGAP5. Left panel: IGV-sashimi plot showing the regulated alternative splicing events and binding sites across mRNA. The read distribution of RASE is plotted in the top panel, and the transcripts of each gene are presented in the bottom panel. Right panel: The schematics depicting the structures of ASEs. RNA-seq validation of ASEs is shown in the bottom right panel. Error bars represent mean ± SEM. **P-value < 0.01. (C) FSCN1 regulates alternative splicing of WTAP. Left panel: IGV-sashimi plot showing the regulated alternative splicing events and binding sites across mRNA. The read distribution of RASE is plotted in the top panel and the transcripts of each gene are shown below. Right panel: The schematics depicting the structures of ASEs. RNA-seq validation of ASEs are shown at the bottom of the right panel. Error bars represent mean ± SEM. **P-value < 0.01. (D) FSCN1 regulates alternative splicing of RECQL5. Left panel: IGV-sashimi plot showing the RASEs and binding sites across mRNA. The read distribution of RASE is plotted in the top panel and the transcripts of each gene are presented in the bottom panel. Right panel: The schematics depicting the structures of ASEs. RNA-seq validation of ASEs is shown at the bottom right panel. Error bars represent mean ± SEM. **P-value < 0.01. [file peerj-11-16526-s008.pdf]

A

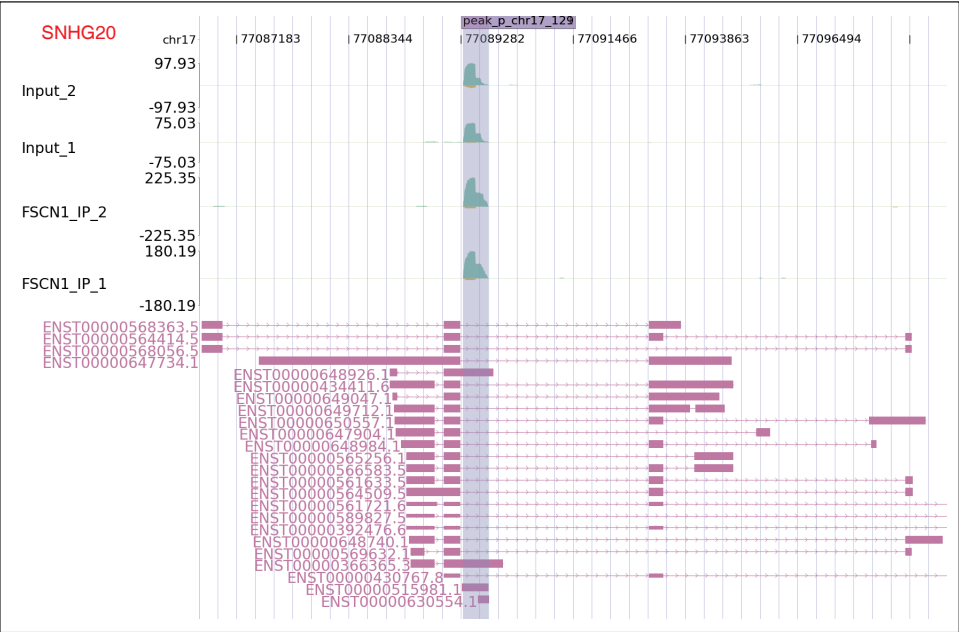

Supplement: Figure S9 — (A) FSCN1 binding peak genes of SNHG20. IGV-sashimi plot showing the peaks reads and binding sites across mRNA; the green and red panels represent the position of peaks. The read distribution of the bound gene is plotted in the top panel, and the transcripts of each gene are presented in the bottom panel. [file peerj-11-16526-s009.pdf]

A

## Overall Survival

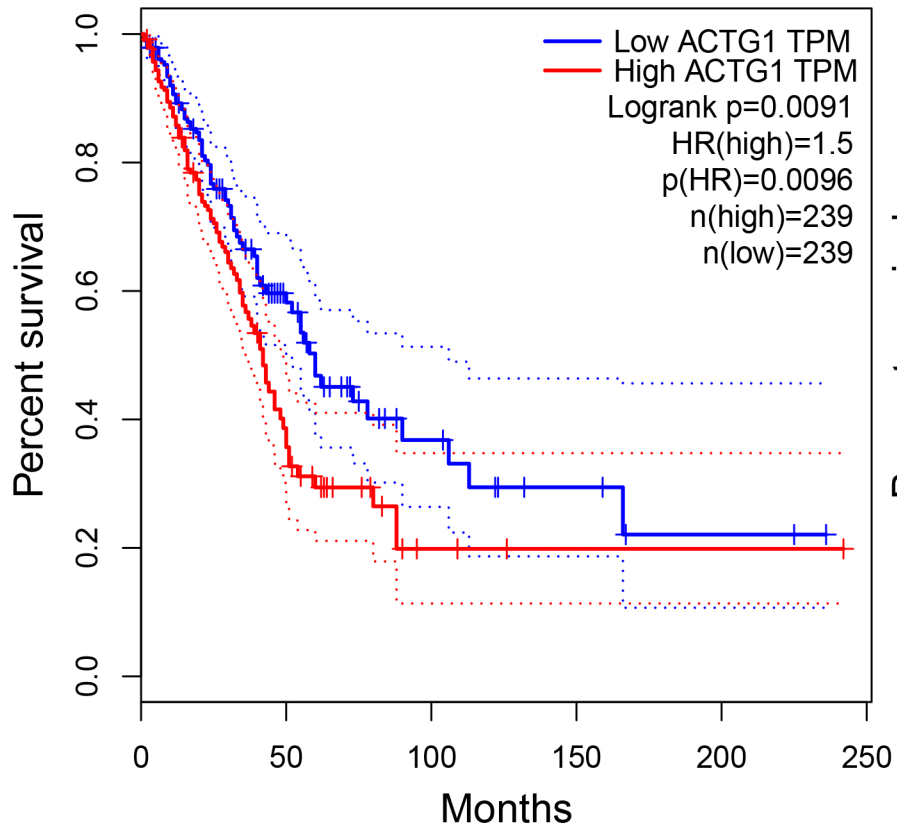

B

## Overall Survival

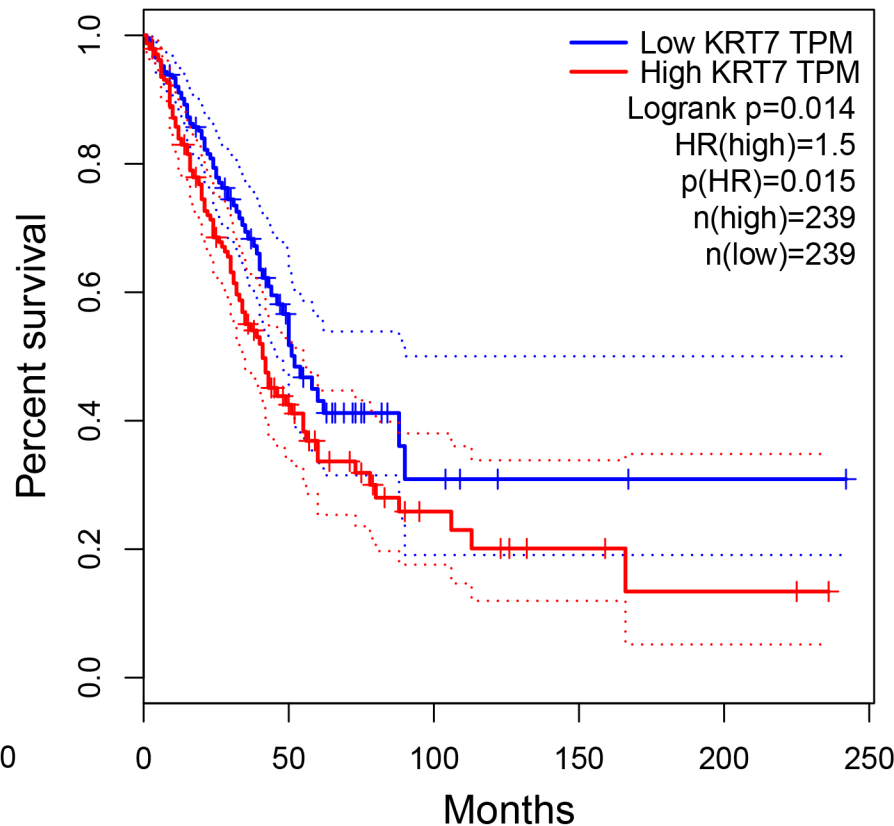

Supplement: Figure S10 — (A) Kaplan –Meier plot showing the prognosis difference between LUAD patients with high and low ACTG1 expression levels. (B) Kaplan–Meier plot showing the prognosis difference between LUAD patients with high and low KRT7 expression levels. [file peerj-11-16526-s010.pdf]

A

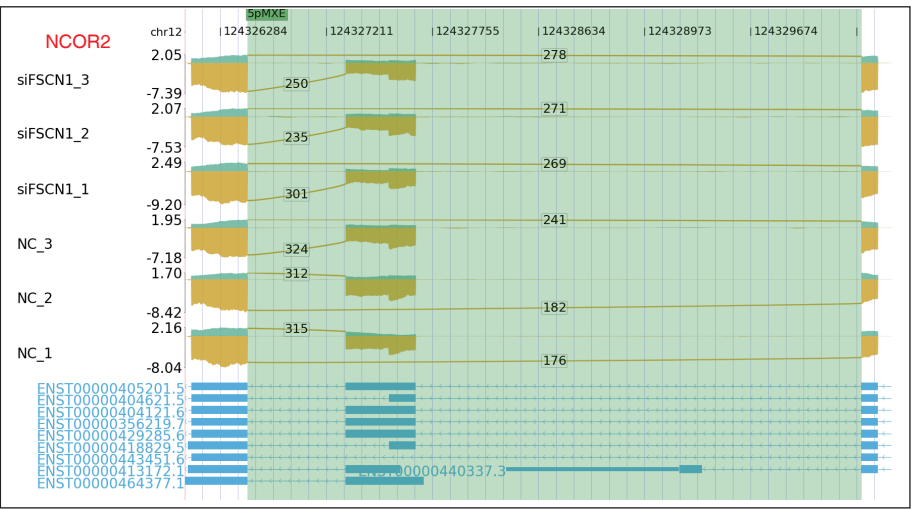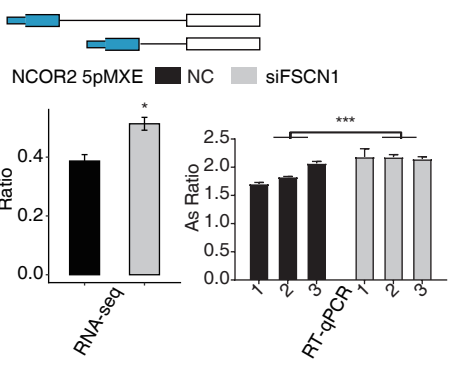

B

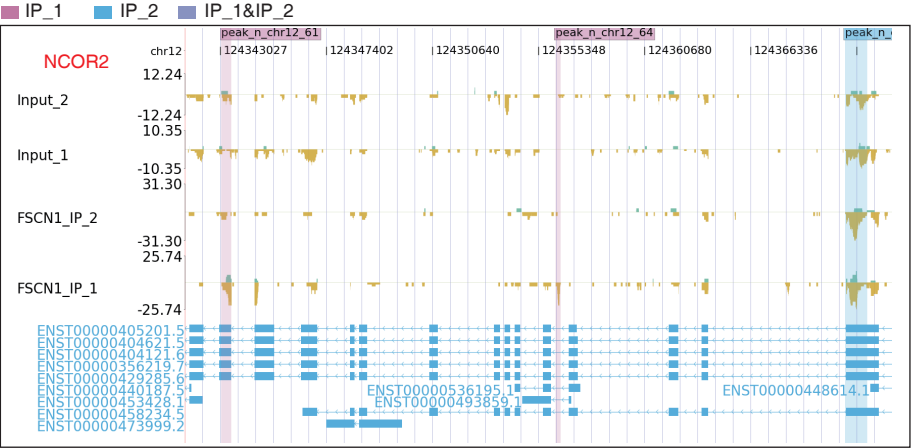

Supplement: Figure S11 — (A) FSCN1 regulates alternative splicing of NCOR2. Left panel: IGV-sashimi plot showing the regulated alternative splicing events (RASEs) and binding sites across mRNA. The read distribution of RASE is plotted in the top panel, and the transcripts of each gene are presented in the bottom panel. Right panel: schematics depicting the structures of ASEs. RNA-seq validation of ASEs is shown at the bottom right panel. RNA-seq validation of ASEs is shown at the bottom right panel. Reverse transcription qPCR validation of ASEs regulated by FSCN1 in cancer cells; black bars denote the control group, and grey bars denote the FSCN1-silenced group. Error bars represent mean ± SEM. *P-value < 0.05, ***P-value < 0.001. (B) FSCN1 binding peak genes of NCOR2. IGV-sashimi plot showing the peaks reads and binding sites across mRNA, the green and red panels represent the position of peaks. The read distribution of the bound gene is plotted in the top panel, and the transcripts of each gene are presented in the bottom panel. [file peerj-11-16526-s011.pdf]

A

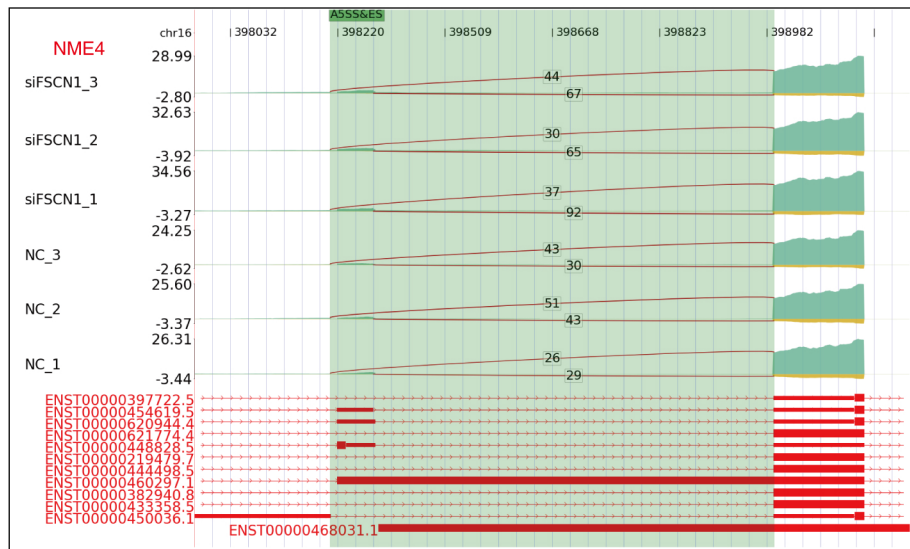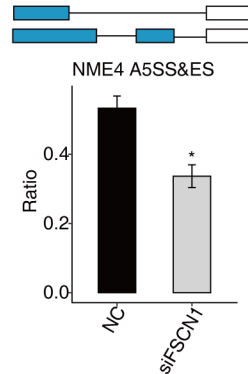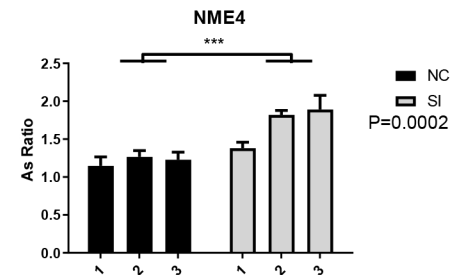

B

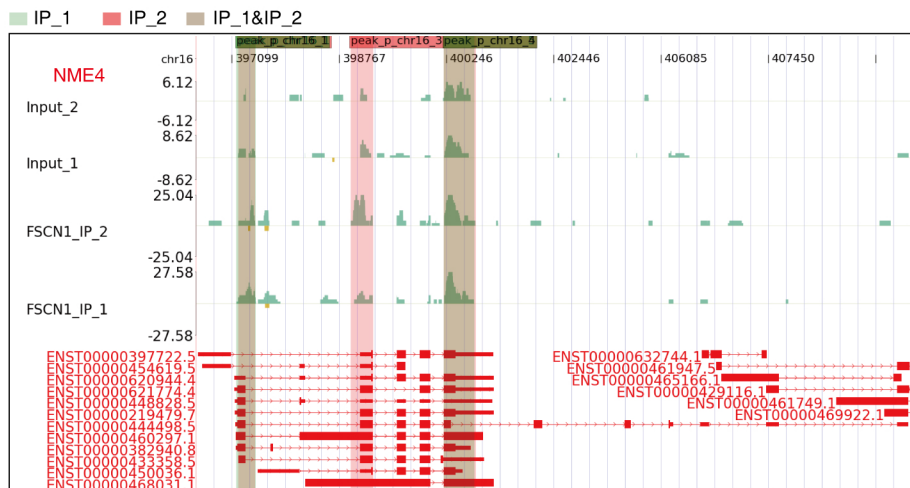

Supplement: Figure S12 — (A) FSCN1 regulates alternative splicing of NME4. Left panel: IGV-sashimi plot showing the regulated alternative splicing events (RASEs) and binding sites across mRNA. The read distribution of RASE is plotted in the top panel, and the transcripts of each gene are presented in the bottom panel. Right panel: schematics depicting the structures of ASEs. RNA-seq validation of ASEs is shown at the bottom right panel. RNA-seq validation of ASEs are shown at the bottom right panel. Reverse transcription qPCR validation of ASEs regulated by FSCN1 in cancer cells; black bars denote the control group, and grey bars denote the FSCN1-silenced group. Error bars represent mean ± SEM. * P-value < 0.05, ***P-value < 0.001. (B) FSCN1 binding peak genes of NME4. IGV-sashimi plot showing the peak reads and binding sites across mRNA; the green and red panels represent the position of peaks. The read distribution of the bound gene is plotted in the top panel, and the transcripts of each gene are presented in the bottom panel. [file peerj-11-16526-s012.pdf]
