# Supplementary material for: Fascin actin-bundling protein 1 regulates non-small cell lung cancer progression by influencing the transcription and splicing of tumorigenesis-related genes
Source: PeerJ. 2023 Dec 5;11:e16526. doi: 10.7717/peerj.16526 (PMC10704988; doi:10.7717/peerj.16526)
Supplement: Table S1 [file peerj-11-16526-s013.docx]

| **Table S1 Primers Information** | |
| --- | --- |
| Gene | Sequence (5'-3') |
| hum-GAPDH-F | GGTCGGAGTCAACGGATTTG |
| hum-GAPDH-R | GGAAGATGGTGATGGGATTTC |
| FSCN1-F | AGGGGACTCAGAGCTCTTCC |
| FSCN1-R | TGCCTGTGGAGTCTTTGATG |
| UBE2S-F | CCTCTTGAGCACGTTGAC |
| UBE2S-R | AGGTCTGTTCCGCATGAA |
| KRT18-F | GCCTACAAGCCCAGATTG |
| KRT18-R | AGCTCCTCTCGGTTCTTC |
| KRT7-F | GGCTCCTGAAGGCTTATTC |
| KRT7-R | GACTGTCTCACTGTCTTGG |
| PDE3A-F | TACCAGAACAAATCTTTCCG |
| PDE3A-R | CCTGAGTCTCATGCCTTT |
| ACTG1-F | AGAATCGAGAATTGCGTACA |
| ACTG1-R | GAAAGTCCAAGCCGTAGG |
| NME4-AS-F | CTATAGGAAGGAGGGCCCTC |
| NME4-M-F | GGCTCACCTGGAGGGCCCTC |
| NME4-M/AS-R | CATCTTCATCCCCACCAGCGT |
| SGO1-M/AS-F | CCACTGCTGGGCTTGCTTTA |
| SGO1-AS-R | ACTCCTACCACTACACCACC |
| SGO1-M-R | CAAATAGAAGCTACACCACC |
| WTAP-M/AS-F | TTGCCCAACTGAGATCAACA |
| WTAP-AS-R | CATCAGGCCTCTATCAGGCG |
| WTAP-M-R | CCCTGTTTGGCTATCAGGCG |
| EEF1D-M/AS-F | TCTGCTCGTAGAATCTCCTTT |
| EEF1D-AS-R | TGAGACCCAACAGAAAAATG |
| EEF1D-M-R | TTAGACGCTACAGAAAAATG |
| NCOR2-M/AS-F | CACTGAGGAGACAGAGGGTGGC |
| NCOR2-M-R | TCACCTCGCCAGGTGGCGGC |
| NCOR2-AS-R | TCACCGGAACAGGTGGCGGC |
