# Supplementary material for: Fascin actin-bundling protein 1 regulates non-small cell lung cancer progression by influencing the transcription and splicing of tumorigenesis-related genes
Source: PeerJ. 2023 Dec 5;11:e16526. doi: 10.7717/peerj.16526 (PMC10704988; doi:10.7717/peerj.16526)
Supplement: Table S2 [file peerj-11-16526-s014.docx]

| **Table S2 Sample Description** | | |
| --- | --- | --- |
| Sample ID | Sample name | Sample Description |
| 1 | NC-1 | the scramble (NC) siRNA |
| 2 | NC-2 |  |
| 3 | NC-3 |  |
| 4 | Si 1-1 | Si FSCN1-556 |
| 5 | Si 1-2 |  |
| 6 | Si 1-3 |  |
| 7 | Si 2-1 | Si FSCN1-1122 |
| 8 | Si 2-2 |  |
| 9 | Si 2-3 |  |
| 10 | Si 3-1 | Si FSCN1-1926 |
| 11 | Si 3-2 |  |
| 12 | Si 3-3 |  |
